# *C. elegans* TAT-6, a putative aminophospholipid translocase, is expressed in sujc cells in the hermaphrodite gonad.

**DOI:** 10.17912/micropub.biology.000495

**Published:** 2021-11-04

**Authors:** Lars Nilsson, Shapour Rahmani, Simon Tuck

**Affiliations:** 1 UCMM, Umeå University, Sweden

## Abstract

In healthy eukaryotic cells, the two leaflets that make up plasma membranes are highly asymmetric with respect to the lipids they contain. In both unicellular eukaryotes and metazoans, the asymmetry in the distribution of aminophospholipids is maintained by P4-family transmembrane ATPases, which catalyze the movement of selected phospholipids from the outer leaflet to the inner. *C. elegans *has six P4-family ATPases, TAT-1 – TAT-6. TAT-1 – TAT-5 are expressed in many tissues and cells. Here we report that, in contrast, TAT-6 is much less broadly expressed and that, within the somatic gonad, expression of TAT-6 reporters is restricted to the spermathecal-uterine core cell (sujc) cells.

**Figure 1. tat-6 is expressed in sujc cells:**
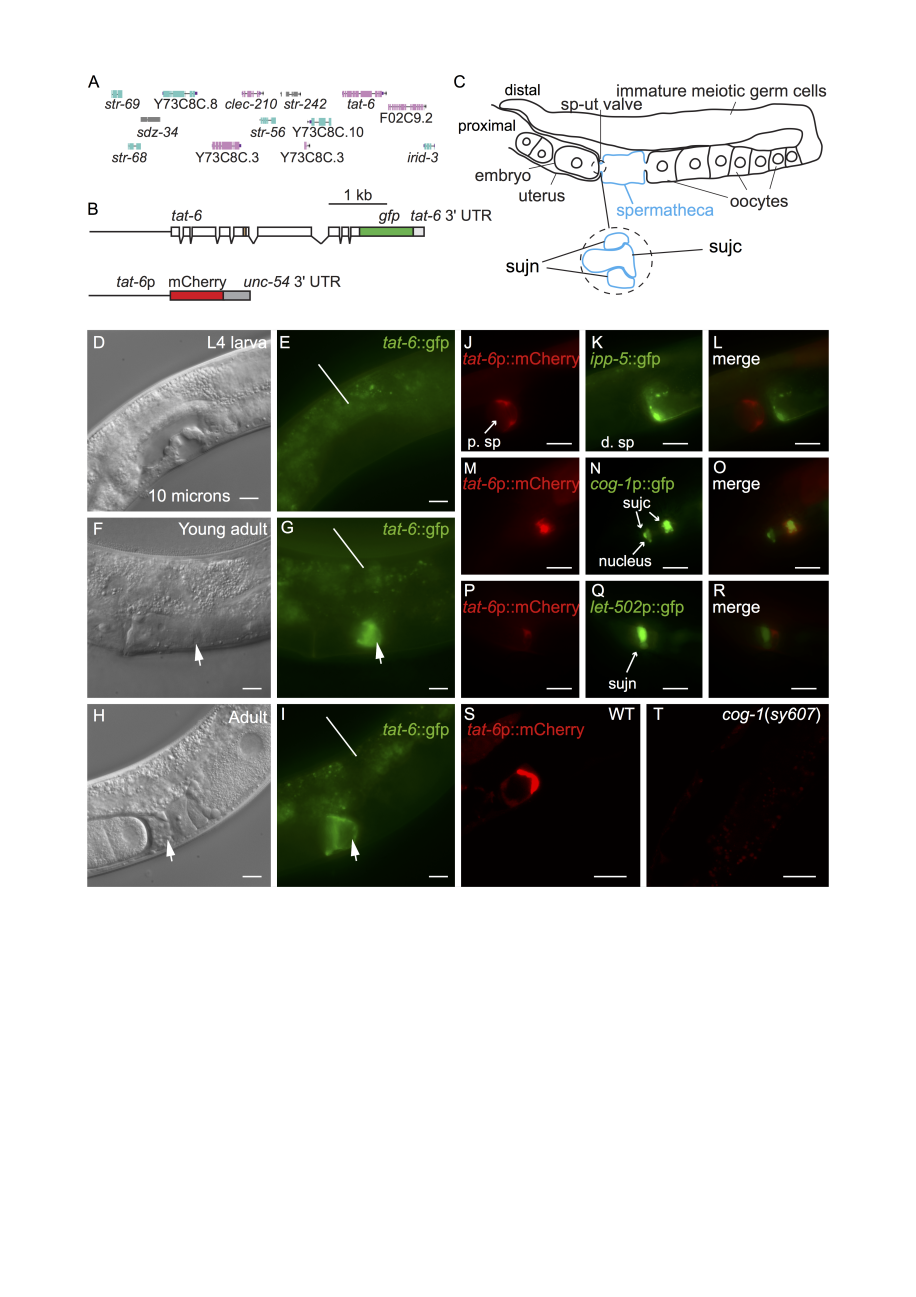
A. The *tat-6* chromosomal region. The genes shown are those present in the fosmid used to generate the *tat-6*::gfp reporter shown in B. B. Schematics of the *tat-6* reporters used in this study. Boxes represent exons. The upper construct encodes a TAT-6::GFP fusion protein in which GFP is fused to the whole of TAT-6. The beige-colored box indicates the part of TAT-6 containing the P-type ATPase motif. The reporter gene was generated by inserting gfp-encoding sequences into the fosmid (Sarov *et al.* 2012). The fosmid also contains the genes (shown in A) to the left and right of *tat-6* in the *C. elegans* genome as well as intragenic sequences (Sarov *et al.* 2012). The *tat-6*::gfp reporter has the *tat-6* 3ʹ untranslated region (UTR). The lower construct is a transcriptional reporter in which mCherry expression is driven by a 1.4 Kb fragment from the *tat-6* promoter. This construct contains the 3ʹ-UTR of the *unc-54* gene. C. A schematic showing one arm of the gonad in an adult hermaphrodite. The spermatheca is highlighted in blue. ‘sp-ut valve’ indicates the position of the spermathecal-uterine valve. The expanded region shows a schematic cross section of the valve as it exists prior to ovulation. In such animals, the sujc syncytium occupies the core of the valve and is surrounded by a toroidal syncytium, sujn (Kimble and Hirsh 1979). During the first ovulation, sujc is displaced from the core of the valve. D-I, micrographs of the mid-body regions of hermaphrodite worms harboring the *tat-6*::GFP transgene shown in B. In D, F and H the worms were viewed with Nomarksi differential interference contrast (DIC) optics; E, G and I show the same worms viewed with fluorescence optics. In F and H the arrows indicate the position of the spermathecal-uterine valve. The uterus is to the left of the arrows, the spermatheca to the right. In G and I the arrows indicate GFP fluorescence in the gonad. The lines in E, G and I indicate background autofluorescence from the intestine. H shows a hermaphrodite that contained fertilized eggs in the uterus; the egg in closest proximity to the spermatheca is partially enveloped by material containing the TAT-6::GFP fusion protein. J,K,L. Fluorescent micrographs of an adult hermaphrodite carrying *tat-6*p::mCherry and *ipp-5*::GFP transgenes. The worms were mounted for photography so that the uterus was to the left. The *ipp-5*::GFP transgene is expressed in distal spermathecal cells (d. sp) at the junction with the ovary (Bui and Sternberg 2002). Note the lack of overlap between the mCherry and GFP fluorescence signals. M,N,O. Fluorescence micrographs of a hermaphrodite harboring *tat-6*p::mCherry and *cog-1*p::GFP transgenes. The *cog-1* transgene is expressed in the sujc syncytium, which forms the core of the spermathecal-uterine valve (Palmer *et al.* 2002). To the right in the panels, the mCherry and GFP signals overlap. Note that while the distal-most part of the sujc syncytium is firmly within the center of the core, the sujc cell nuclei protrude into the uterus and are almost 10 microns from the distal part of the cell (Palmer *et al.* 2002). While some GFP encoded by the *cog-1*p::GFP transgene is nuclear, the mCherry signal is strongest in the distal part of the syncytium. P,Q,R. Fluorescent micrographs of a hermaphrodite harboring *tat-6*p::mCherry and *let-502*p::GFP transgenes. The *let-502*p::GFP transgene is strongly expressed in sujn cells (Wissmann *et al.* 1999). S,T. Confocal fluorescence micrographs of adult hermaphrodites harboring the *tat-6*p::mCherry transgene. S shows an otherwise wild-type hermaphrodite; T shows a *cog-1* mutant. Scale bars in all panels indicate 10 microns.

## Description

*tat-1* and *tat-5* are both very broadly expressed in *C. elegans* (Lyssenko *et al.* 2008, Ruaud *et al.* 2009, Chen *et al.* 2010, Wehman *et al.* 2011). *tat-2*, *tat-3* and *tat-4* are also expressed in many cells and tissues although apparently not ubiquitously (Lyssenko *et al.* 2008). No reports exist presently describing the pattern in which *tat-6* is expressed. We generated multiple transgenic lines containing a construct encoding the entire TAT-6 protein fused to GFP (Fig. 1A,B). The construct contained *tat-6* promoter sequences, all exons and introns as well as intragenic sequences to the left and right of the *tat-6* coding region (Fig. 1A,B) (Sarov *et al.* 2012). Expression was seen in cells in the head and tail but in the center of the worm, expression was restricted to a proximal part of the gonad (Fig. 1C,F,G,H,I). Expression in the gonad was absent in early and mid-L4 stage worms but was robust in young adults (Fig. 1D,E,F,G). Prior to ovulation, fluorescence was seen in a region at the junction between the spermatheca and the uterus (Fig. 1C,F,G). In hermaphrodites in which ovulation had occurred, GFP fluorescence was seen partially surrounding the distal-most egg in the uterus (Fig.1H,I). The valve forming the junction between spermatheca and the uterus consists of a toroidal syncytium, sujn, and a core cell syncytium, sujc, which initially occupies the center of the valve (Fig. 1C) (Kimble and Hirsh 1979, Lints and Hall 2013). During the first ovulation, the core is displaced by the passage of the newly fertilized egg from the spermatheca to the uterus (Kimble and Hirsh 1979). The change in the distribution of TAT-6::GFP fluorescence we observed during the first ovulation suggested that TAT-6 might be expressed in sujc. Since existing GFP markers for cells in the spermatheca were available, to determine in which cells *tat-6* was expressed, we first constructed strains containing a *tat-6*p::mCherry transcriptional reporter (Fig. 1B). The marker was expressed in the same way as the GFP reporter (Fig. 1S). In a strain containing the *tat-6*p::mCherry reporter and a reporter in which GFP expression was driven by promoter sequences from the *cog-1* gene active in sujc (Palmer *et al.* 2002), the mCherry and GFP signals were seen in the same cell (Fig. 1M,N,O). In contrast, in a strain containing the *tat-6*p::mCherry reporter and an *ipp-5*p::gfp reporter, which is expressed in distal spermathecal cells that form part of the junction with the ovary (Bui and Sternberg 2002), the two fluorescent signals did not overlap (Fig. 1J,K,L). Similarly, in a strain containing the *tat-6*p::mCherry reporter and an *let-502*p::gfp reporter, which is strongly expressed in sujn cells (Wissmann *et al.* 1999), the mCherry signal was adjacent to the strong GFP expression rather than coincident with it (Fig. 1P,Q,R). To further verify the identity of the cells expressing the *tat-6* reporters, we crossed the *tat-6*p::mCherry transgene into a *cog-1*(*sy607*) mutant background. *cog-1* encodes a GTX/Nkx6.1 homeodomain transcription factor; in *cog-1*(*sy607*) mutant hermaphrodites, cells having the morphology of sujc cells are absent (Palmer *et al.* 2002). *cog-1* is not expressed in sujn cells (Palmer *et al.* 2002). Consistent with the results with the fluorescent markers, no *tat-6* reporter expression was seen in the gonad in the *cog-1*(*sy607*) mutant (Fig. 1S,T).

We do not presently know the function of TAT-6 in sujc. Indeed, the function of the sujc cells themselves is not presently known: ablation of sujc cells in the mid-L4 stage causes only a weak effect on brood size (Palmer *et al.* 2002) (although an earlier function for sujc cells in morphogenesis of the spermathecal-uterine junction has not been ruled out (Palmer *et al.* 2002)). Genetic research in *Saccharomyces cerevisiae* has revealed that all five P4-family ATPases in this organism, through their actions as phospholipid translocases, promote one or more vesicle transport events in the endosomal or secretory pathways (Hankins *et al.* 2015, Pomorski and Menon 2016, Yang *et al.* 2018). It is thought that aminophospholipid translocases promote membrane bending that occurs during the formation of transport vesicles (Hankins *et al.* 2015, Pomorski and Menon 2016, Yang *et al.* 2018). *C. elegans tat-1* is required for correct vesicle transport within the endolysosomal system (Ruaud *et al.* 2009, Chen *et al.* 2010, Nilsson *et al.* 2011). *C. elegans tat-5* is required for endosome to Golgi trafficking of MIG-14 (a *C. elegans* Wntless homologue) in the QL neuroblast (McGough *et al.* 2018), and to suppress the formation of extracellular vesicles in the embryo (Wehman *et al.* 2011, Beer *et al.* 2018). Thus, it is possible that TAT-6 promotes one or more vesicle transport event within sujc. It is worth noting that the plasma membrane of sujc cells is unusual in being highly convoluted (Kimble and Hirsh 1979, Lints and Hall 2013). Although it is not known how the extensive folding of the membrane arises, cell autonomous processes that promote membrane folding in sujc cells have not been ruled out. Finally, our studies also shed light on what happens to material from the sujc cells following ovulation. The fate of these cells after the core of the spermathecal-uterine valve has been displaced is presently not known. The fact that some TAT-6::GFP fusion protein (and mCherry) remains in the uterine epithelium even in older hermaphrodites indicates that at least a part of the sujc cells is retained following ovulation.

## Methods

Standard methods were used in the maintenance of *C. elegans* worms. Clone I16253250892533I A08 (Sarov *et al.* 2012) was used to generate *svEx940*, the extrachromosomal array from which the integrated array *svIs144* was derived; pVB652 was used to generate *svEx967* and *svEx968*. Transgenic strains were generated by microinjection (Fire 1986). The DNA clones were microinjected at a concentration of 50 ng/μl. *svIs144* was derived from *svEx940* by γ-irradiation. Micrographs were made with DM6000 B and DMRB compound microscopes (Leica); confocal micrographs were made with an A1 confocal microscope (Nikon).

## Reagents

**Table:** 

**Strain**	**Genotype**	**Available from**
VB3030	*unc-119*(*ed3*op); *svIs144*[*tat-6*p::*tat-6*::GFP *unc-119*(+)]	This work
VB3041	*unc-4*(*e120*); *svEx967*[*tat-6*p::mCherry *unc-4*(+)]	This work
PS3747	*ipp-5*(*sy605*); *syEx429*[*ipp-5*::GFP *rol-6*(*su1006*)]	CGC
PS3662	*syIs63*[*cog-1*p::GFP]	CGC
VB2238	*svEx968*[*tat-6*p::mCherry]; *syEx429*[*ipp-5*::GFP *rol-6*(*su1006*)]	This work
VB3122	*syIs63*[cog*-1*p::GFP]; *svEx968*[*tat-6*p::mCherry *unc-4*(+)]	This work
HR606	*sbEx136*[*let-502p*::GFP *rol-6*(*su1006*)]	CGC
VB3123	*sbEx136*[*let-502p*::GFP *rol-6*(*su1006*)]; *svEx968*[*tat-6*p::mCherry *unc-4*(+)]	This work
VB3357	*cog-1*(*sy607*); *svEx967*[*tat-6*p::mCherry *unc-4*(+)]	This work
